# Neonatal Screening for MPS Disorders in Latin America: A Survey of Pilot Initiatives

**DOI:** 10.3390/ijns6040090

**Published:** 2020-11-13

**Authors:** Francyne Kubaski, Inês Sousa, Tatiana Amorim, Danilo Pereira, Joe Trometer, Alexandre Souza, Enzo Ranieri, Giulia Polo, Alberto Burlina, Ana Carolina Brusius-Facchin, Alice Brinckmann Oliveira Netto, Shunji Tomatsu, Roberto Giugliani

**Affiliations:** 1 Postgraduate Program in Genetics and Molecular Biology, Universidade Federal do Rio Grande do Sul (UFRGS), Porto Alegre 90040-060, Brazil; rgiugliani@hcpa.edu.br; 2 Medical Genetics Service, Hospital de Clínicas de Porto Alegre (HCPA), Porto Alegre 90035-903, Brazil; afacchin@hcpa.edu.br (A.C.B.-F.); alicenetto@hcpa.edu.br (A.B.O.N.); 3 Instituto Nacional de Genética Médica Populacional (iNaGeMP), Porto Alegre 90035-003, Brazil; 4 Associação de Pais e Amigos dos Excepcionais (APAE) Salvador, Salvador 41830-141, Brazil; ines.sousa@apaesalvador.org.br (I.S.); tatiana.amorim@apaesalvador.org.br (T.A.); 5 Department of Research and Innovation, Innovatox, São Paulo 06455-020, Brazil; danilopereira@innovatox.com.br; 6 PerkinElmer, Waltham, MA 02451, USA; joe.trometer@perkinelmer.com; 7 PerkinElmer, São Paulo 02518-080, Brazil; alexandre.souza@perkinelmer.com; 8 SA Pathology, Women’s and Children’s Hospital, Adelaide, SA 5006, Australia; Enzo.Ranieri@sa.gov.au; 9 Division of Inherited Metabolic Diseases, Department of Diagnostic Services, University Hospital of Padua, 35129 Padua, Italy; giulia.polo@aopd.veneto.it (G.P.); alberto.burlina@unipd.it (A.B.); 10 Department of Biological Sciences, Universidade Federal do Rio Grande do Sul (UFRGS), Porto Alegre 90040-060, Brazil; 11 Department of Orthopedics and Biomedical, Nemours/Alfred I. duPont Hospital for Children, Wilmington, DE 19803, USA; Shunji.Tomatsu@nemours.org

**Keywords:** mucopolysaccharidosis, newborn screening, glycosaminoglycans, enzyme assays, tandem mass spectrometry, fluorimetry

## Abstract

Newborn screening enables the diagnosis of treatable disorders at the early stages, and because of its countless benefits, conditions have been continuously added to screening panels, allowing early intervention, aiming for the prevention of irreversible manifestations and even premature death. Mucopolysaccharidoses (MPS) are lysosomal storage disorders than can benefit from an early diagnosis, and thus are being recommended for newborn screening. They are multisystemic progressive disorders, with treatment options already available for several MPS types. MPS I was the first MPS disorder enrolled in the newborn screening (NBS) panel in the USA and a few other countries, and other MPS types are expected to be added. Very few studies about NBS for MPS in Latin America have been published so far. In this review, we report the results of pilot studies performed in Mexico and Brazil using different methodologies: tandem mass spectrometry, molecular analysis, digital microfluidics, and fluorimetry. These experiences are important to report and discuss, as we expect to have several MPS types added to NBS panels shortly. This addition will enable timely diagnosis of MPS, avoiding the long diagnostic odyssey that is part of the current natural history of this group of diseases, and leading to a better outcome for the affected patients.

## 1. Introduction

Newborn screening (NBS) allows for a reduction of morbidity and mortality of selected treatable disorders within the first few days of life, enabling early treatment of the newborns [1]. The beginning of NBS was in the 1960s, with the pioneering work of Robert Guthrie and Ada Susi, who have implemented a screening test for phenylketonuria (PKU) [2]. The benefits of NBS soon became clear, and a growing number of conditions were continuously added to screening panels to bring the benefits of early detection and early intervention to more and more babies [3]. This impact is so significant that the Centers for Disease Control and Prevention (CDC) considers NBS one of the ten great public health achievements of the 21st century [4], as it may proportion to the detected baby interventions and benefits such as prevention of developmental delay, severe disability, or even premature death [3].

Mucopolysaccharidoses (MPS) are progressive disorders that can potentially lead to death within the first decades of life due to severe clinical manifestations, such as neurological impairment, skeletal abnormalities, as well as pulmonary and cardiac problems [5,6]. Although there is still no cure for MPS, there are several treatment options already approved that could benefit most MPS patients. Diagnosis based on clinical suspicion usually takes a few years to be achieved [7], and there is already robust evidence about the benefits of early intervention [8,9]. Thus, advocacy for the addition of newborn screening for MPS led to the addition of MPS I by the Advisory Committee on Heritable Disorders in Newborns and Children (ACHDNC) to the Recommended Uniform Screening Panel (RUSP) in February of 2016 [10,11]. Missouri was the first state to screen for MPS I [12], followed by several other states, and the state of Illinois has also started the screening of MPS II in 2017 [13,14,15], followed by Missouri in 2019.

A pilot study for the screening of MPS I was performed in Taiwan [16] in 2008, followed by a pilot study for MPS I and II [17], and then by a large-scale NBS program of MPS I, MPS II, and MPS VI in 2015 [18]. A pilot study for MPS IVA was also performed in Taiwan in 2013 [19]. Another pilot study was performed in the Tuscany and Umbria regions of Italy for MPS I [20], and also in the northeast of Italy [21,22]. MPS I and II NBS pilot studies were performed in Osaka [20,23], and a small feasibility study for NBS of MPS II was performed in the Netherlands [24].

Despite these major achievements, a wide-scale inclusion of lysosomal disorders (LSDs), such as MPS in the NBS program, is still moving slowly. The UK National Screening Committee (UK-NSC) does not currently recommend the inclusion of MPS I in their systematic population screening program [25], and this is no different in Latin America, where there is a considerable variation in the screening panels among different countries, and where standard neonatal screening is still not routine in several nations [26]. Comprehensive data about newborn screening in Latin America can be found in several surveys [1,27,28]. This paper provides a review of the pilot newborn screening programs for MPS performed in Latin America.

## 2. Methods and Results

Very few studies of NBS for MPS were performed in Latin America so far. We will describe below the methods and results of studies already published and the novel results from our group that are being reported for the first time.

### 2.1. Pilot Study for Six LSDs (Including MPS I) in Mexico

A pilot study for the screening of 6 LSDs has been performed in Mexico from 2012, aiming to properly identify newborns with LSDs that could benefit from the introduction of early treatment, and also to estimate the incidence of these disorders in Mexico.

This study has evaluated the use of a multiplex platform for the detection of Fabry disease, Gaucher disease, Krabbe disease, MPS I, acid sphingomyelinase deficiency (ASMD), and Pompe disease. In a report of 2017, 20,018 dried blood spots (DBS) were analyzed by tandem mass spectrometry (MS/MS), with 20 patients reported as positives and submitted to confirmatory testing with enzyme assays and DNA analysis.

The confirmed diagnoses were as follows: five with Fabry disease, two with ASMD, and two with MPS I. Both MPS I patients were compound heterozygotes (p.Val322Glu/p.Arg621Ter, p.Val322Glu/p.Ser234Thr) [29]. The combined use of an enzyme assay with biomarkers can decrease the false-positive rates.

### 2.2. Targeted Newborn Screening for MPS VI in Northeast Brazil

In a remote city known as Monte Santo (estimated population of 52,338), located in the state of Bahia (Northeast Brazil), a very high frequency of MPS VI (1:5000 inhabitants) was found, attributed to the founder effect and endogamy [30]. 13 cases of MPS VI have been identified in this town, all of whom have the same pathogenic variant (p.His178Leu) [30].

Due to the high frequency, the availability of specific treatment with enzyme replacement therapy (ERT), and the evidence that shows the benefits of early intervention [8], a pilot study was proposed to be carried out in this area. The pilot study has started with the measurement of arylsulfatase B (ARSB) in DBS samples with a fluorometric method that was adapted for microplates [31]. However, due to the high rate of false positives, and to the fact that all patients previously identified carry the same pathogenic variant, the screening method was changed to molecular genetics, with the detection of the common variant by real-time PCR in DBS [32].

From 2011 to 2017, 3903 newborns were analyzed, and 67 (1.72%) were found to be carriers for p.His178Leu. Although no affected patients were identified, the carrier rate allowed an estimation of the disease incidence as 1:16,000 per live births in the area [32]. This pilot study was part of a community genetics program that involved educational activities in the community and genetic counseling to the population at risk [32].

### 2.3. Pilot Program for Four LSDs (Including MPS I) in a Private Laboratory in Brazil

A pilot study for four LSDs was performed by a private Brazilian laboratory in 10,527 newborns. The conditions tested were as follows: Fabry disease, Gaucher disease, MPS I, and Pompe disease by digital microfluidics (DMF) [33].

Four samples had activities below the cutoff (one for Gaucher, two for MPS I, and one for Pompe) [33]. The two screened positives for MPS I had normal urinary GAGs by dimethylmethylene blue (DMB) and a normal GAG pattern after eletroctrophoresis [34]. One newborn was a compound heterozygote for a pathogenic variant and a pseudodeficiency allele (p.Gly84Ala/p.His82Gln), respectively, and the other was a heterozygote for p.Trp402Ter [34]. Although no case of MPS I was confirmed, the methodology was considered sensitive to detect low activity alpha-iduronidase (IDUA) in DBS.

### 2.4. Pilot Program for Five LSDs (Including MPS I and MPS VI) in a Research Laboratory in Brazil

Another pilot study was reported in Brazil for MPS I, MPS VI, Fabry disease, Gaucher disease, and Pompe disease using a fluorimetric assay in 834 newborns. The method was adapted in-house for microplates, but the results indicated a high proportion of false positives, especially for MPS I (46 for MPS I, 7 for MPS VI, 0 for Fabry, 14 for Gaucher, and 25 for Pompe). Urinary GAGs were measured by DMB to investigate the babies who tested positive for MPS (35 urines for the suspected MPS I out of 46 and 2 urines of the 6 suspected MPS VI), but all babies with available urine samples had normal GAG results [35].

### 2.5. Pilot Newborn Screening Program for Six LSDs in a Research Laboratory in Brazil

Lastly, our group is currently performing an NBS pilot study for 6 LSDs (Fabry disease, Gaucher disease, Krabbe disease, MPS I, ASMD, and Pompe disease) by MS/MS, with the target to screen 20,000 newborns.

**Figure 1:**
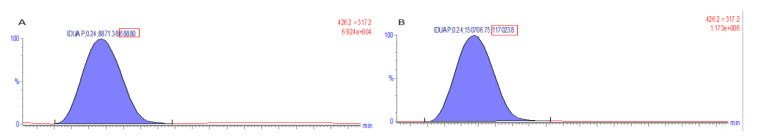
The alpha-iduronidase (IDUA) activities in a selected newborn with low IDUA and a newborn with normal levels of IDUA. (**A**) IDUA product peak area (red box) of 68,880, resulting in a low IDUA of 0.22 nmoL/h/mL; (**B**) IDUA product peak area (red box) of 1,170,238, resulting in an activity of 4.7 nmoL/h/mL in a random newborn sample.

Currently, 11,808 newborns have been analyzed, with two cases of Fabry disease and two cases of Pompe disease detected so far. In one baby, a low IDUA activity was reported (0.22 nmoL/h/mL; cutoff ≤ 0.31 nmoL/h/mL; daily median for general newborns: 5.18 nmoL/h/mL) (Figure 1).

**Figure 2:**
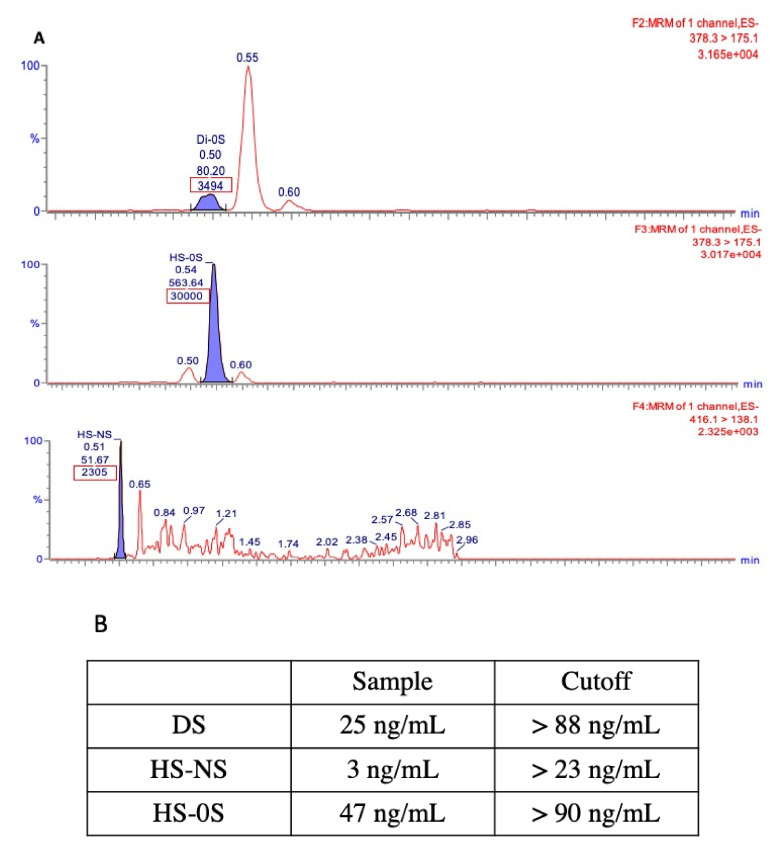
GAG levels in the screen positive sample for IDUA by LC/MS/MS. (**A**) Peak areas (red boxes) of dermatan sulfate (DS), heparan sulfate-0S (HS–0S), and heparan sulfate-NS (HS–NS), respectively. (**B**) GAG quantification in the screened positive sample for IDUA compared to the cutoff for the newborn MPS I samples.

This sample was analyzed for GAGs in the same DBS used for the IDUA quantification to confirm if it was a true positive or a pseudodeficiency. The GAG levels measured in DBS by liquid chromatography tandem mass spectrometry (LC/MS/MS) were below the cutoff, confirming that the low IDUA level was due to a pseudodeficiency (Figure 2).

This DBS sample was also analyzed by next-generation sequencing (NGS) on the Ion S5 System platform (Thermo Scientific™, Waltham, MA, USA) employing a customized and validated panel (Ion AmpliSeq™ Thermo Scientific™), including the *IDUA* gene [36].

Raw sequencing data were analyzed on the bioinformatics platform Ion Torrent Suite and Ion Reporter (Thermo Scientific™) v.5. A list of detected sequence variants, including single nucleotide polymorphisms (SNPs) and small insertions/deletions, was imported into the Ion Reporter™ v5.16.0.1 (Thermo Fisher Scientific™) for annotation.

Alignments were visually verified with the Integrative Genomics Viewer (IGV) v2.3. Mutation Taster [37] and Human Splice Finder [38] were used as pathogenicity prediction tools. The DNA was extracted from the same DBS following an in-house protocol. We have identified three variants in heterozygosity when compared with the reference sequence (NM_000203.4) (Table 1).

**Figure 3:**
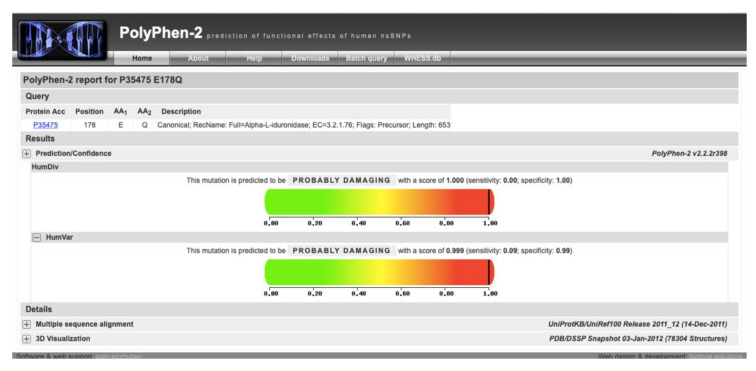
PolyPhen score of 1.000 for the variant p.Glu178Gln. This score predicts the variant to be probably damaging after in silico analysis [40].

Two of the variants found, p.Ala79Thr and p.Thr99Ile, have been reported as causing pseudodeficiency [20]. The third variant has not been described in the literature. For the pathogenicity analysis, in silico programs were used, and predicted this variant as probably damaging (Figure 3). However, according to the American College of Medical Genetics (ACMG) criteria [39], this variant was classified as a variant of uncertain significance (VUS).

Another variant in the same codon has been described in patients with Hurler syndrome (p.Glu178Lys) [41] when combined with another pathogenic variant associated with the severe phenotype. Thus, we conclude that this newborn carries variants associated with pseudodeficiency and carries also a VUS (p.Glu178Gln), which leads to low IDUA activity, but it does not have MPS I and could be classified as having pseudodeficiency. No other sample has been identified with low IDUA until this moment.

## 3. Discussion

Newborn screening has a clear impact on reducing the morbidity and mortality of several treatable disorders, aiding to decrease the disease burden in the patient’s life and its effects on healthcare costs [26]. To the best of our knowledge, until this moment, only Brazil and Mexico have started pilot studies for the implementation of NBS for MPS in Latin America. There are still several limitations for this implementation in Latin American countries: the limited budget of the countries for NBS programs, the limited capacity of NBS laboratories, prioritization of screening for more traditional disorders with higher incidence and best-known screening advantages, lack of awareness about the MPS as to the benefits of their early diagnosis/early treatment, and high cost of therapies for MPS.

Although only MPS I and MPS VI have been included in the pilots performed so far, the availability of methodologies for multiplex screening by LC/MS/MS for MPS II, MPS IIIB, MPS IVA, and MPS VII will allow the testing for these conditions, which have therapies already available or in development, to be added to pilot screening panels soon.

Other limitations that may be faced during the implementation of NBS for MPS, which are phenotypically very heterogeneous, are the challenges of predicting the phenotype, the choice of the most appropriate treatment for each case (ERT versus HSCT, and in a very short future, probably also ERT with fusion proteins, gene therapy, and gene editing), and the cost of the assays. The finding of VUS and pseudodeficiencies may also be a challenge if biomarker measurements (GAG analyses) are not performed [42]. We should also mention the ethical issues related to the identification of carriers and genetic variants in other genes if large gene panels, exomes, or whole-genome sequencing are employed [43].

It is also important to note that enzyme assays by MS/MS or DMF are recommended to be the first-tier test, to avoid false positives [42,44]. A GAG assay in the same DBS could be performed as a second-tier test, but not as a first-tier test due to the high number of false positives (GAGs can also be elevated in other conditions) [45,46,47]. Some centers might prefer to perform sequencing as a second-tier test, but this could lead to unclear conclusions when a VUS is found. Thus, we recommend analyzing the enzyme as a first-tier test followed by GAGs in the same DBS (to avoid recollection and generation of parental anxiety), and then to perform molecular analysis. False positives can also be reduced by the use of post-analytical interpretation tools, such as the Collaborative Laboratory Integrated Reports (CLIRs) [47]. After the confirmatory testing, it is recommended to perform a follow-up with clinical evaluation and to start the appropriate treatment as indicated by the therapeutic guidelines. The interaction between the laboratory group and the clinical follow-up team is essential for the achievement of better outcomes [48].

Another important aspect to recommend is the conduction of pilot studies before the implementation of the screening program because they aid with validation, the establishment of cutoffs, estimation of costs, definition of program algorithms, and also provide useful information for governments to take the appropriate action [49].

## 4. Conclusions

Newborn screening targets conditions that are usually asymptomatic at birth, in which the introduction of therapy before the irreversible disease manifestations have occurred lead to a significant and positive change in its clinical course. New technologies of screening and therapy are driving the evolution of such screening to have more disorders added to the NBS panels. Nowadays, even complex diseases such as MPS become a potential target for NBS, and it now seems that it is just a matter of time before MPS screening will happen on a large scale worldwide, enabling early and timely identification and avoiding the long diagnostic odyssey that is experienced by most patients, whose treatment could only start years after patients become symptomatic and already have irreversible sequelae.

## Figures and Tables

**Table 1:** Next-generation sequencing results of the *IDUA* gene.

Locus	Reference	Genotype	Coverage	cDNA	Protein	dbSNP
Chr4:981673	G	G/A	155	c.235G > A	p.Ala79Thr	rs58037052
Chr4:981734	C	C/T	158	c.296C > T	p.Thr99Ile	rs147490060
Chr4:995294	G	G/C	343	c.532G > C	p.Glu178Gln	rs992336192

## References

[1] Howson C., Cedergren B., Giugliani R., Huhtinen P., Padilla C.D., Palubiak C., Santos M., Schwartz I., Therrell B.L., Umemoto A. (2018). Universal newborn screening: A roadmap for action. Mol. Genet. Metab..

[2] Guthrie R., Susi A. (1963). A simple phenylalanine method for detecting phenylketonuria in large populations of newborn infants. Pediatrics.

[3] González-Irazabal Y., Hernandez de Abajo G., Martínez-Morillo E. (2020). Identifying and overcoming barriers to harmonize newborn screening programs through consensus strategies. Crit. Rev. Clin. Lab. Sci..

[4] Ten Great Public Health Achievements—United States, 2001–2010. https://www.cdc.gov/mmwr/preview/mmwrhtml/mm6019a5.htm.

[5] Neufeld E., Muenzer J. (2001). ; OMMBID; McGraw-Hill Medical. The Mucopolysaccharidoses. The Online Metabolic and Molecular Bases of Inherited Disease.

[6] Muenzer J. (2004). The mucopolysaccharidoses: A heterogeneous group of disorders with variable pediatric presentations. J. Pediatr..

[7] Vieira T., Schwartz I., Muñoz V., Pinto L., Steiner C., Ribeiro M., Boy R., Ferraz V., de Paula A., Kim C. (2008). Mucopolysaccharidoses in Brazil: What happens from birth to biochemical diagnosis?. Am. J. Med. Genet. A.

[8] McGill J.J., Inwood A.C., Coman D.J., Lipke M.L., de Lore D., Swiedler S.J., Hopwood J.J. (2010). Enzyme replacement therapy for mucopolysaccharidosis VI from 8 weeks of age—a sibling control study. Clin. Genet..

[9] Gabrielli O., Clarke L.A., Ficcadenti A., Santoro L., Zampini L., Volpi N., Coppa G. (2016). V 12 year follow up of enzyme-replacement therapy in two siblings with attenuated mucopolysaccharidosis I: The important role of early treatment. BMC Med. Genet..

[10] Kemper A.R., Brosco J., Comeau A.M., Green N.S., Prosser L.A., Ojodu J., Tanksley S., Jones E., Lam K.K. (2015). Newborn Screening for Mucopolysaccharidosis Type 1 (MPS I): A Systematic Review of Evidence Report of Final Findings Final Version 1.1.

[11] Kellar-Guenther Y., McKasson S., Hale K., Singh S., Sontag M.K., Ojodu J. (2020). Implementing Statewide Newborn Screening for New Disorders: U.S. Program Experiences. Int. J. Neonatal Screen..

[12] Hopkins P.V., Campbell C., Klug T., Rogers S., Raburn-Miller J., Kiesling J. (2015). Lysosomal storage disorder screening implementation: Findings from the first six months of full population pilot testing in Missouri. J. Pediatr..

[13] Burton B.K., Hoganson G.E., Fleischer J., Grange D.K., Braddock S.R., Hickey R., Hitchins L., Groepper D., Christensen K.M., Kirby A. (2019). Population-Based Newborn Screening for Mucopolysaccharidosis Type II in Illinois: The First Year Experience. J. Pediatr..

[14] Burton B.K., Hickey R., Hitchins L. (2020). Newborn Screening for Mucopolysaccharidosis Type II in Illinois: An Update. Int. J. Neonatal Screen..

[15] Ames E.G., Fisher R., Kleyn M., Ahmad A. (2020). Current Practices for U.S. Newborn Screening of Pompe Disease and MPSI. Int. J. Neonatal Screen..

[16] Lin S.-P., Lin H.-Y., Wang T.-J., Chang C.-Y., Lin C.-H., Huang S.-F., Tsai C.-C., Liu H.-L., Keutzer J., Chuang C.-K. (2013). A pilot newborn screening program for Mucopolysaccharidosis type I in Taiwan. Orphanet J. Rare Dis..

[17] Chuang C.-K., Lin H.-Y., Wang T.-J., Huang Y.-H., Chan M.-J., Liao H.-C., Lo Y.-T., Wang L.-Y., Tu R.-Y., Fang Y.-Y. (2018). Status of newborn screening and follow up investigations for Mucopolysaccharidoses I and II in Taiwan. Orphanet J. Rare Dis..

[18] Chan M.-J., Liao H.-C., Gelb M.H., Chuang C.-K., Liu M.-Y., Chen H.-J., Kao S.-M., Lin H.-Y., Huang Y.-H., Kumar A.B. (2019). Taiwan National Newborn Screening Program by Tandem Mass Spectrometry for Mucopolysaccharidoses Types I, II, and VI. J. Pediatr..

[19] Chuang C.-K., Lin H.-Y., Wang T.-J., Huang S.-F., Lin S.-P. (2017). Bio-Plex immunoassay measuring the quantity of lysosomal N-acetylgalactosamine-6-sulfatase protein in dried blood spots for the screening of mucopolysaccharidosis IVA in newborn: A pilot study. BMJ Open.

[20] Donati M.A., Pasquini E., Spada M., Polo G., Burlina A. (2018). Newborn screening in mucopolysaccharidoses. Ital. J. Pediatr..

[21] Burlina A.B., Polo G., Salviati L., Duro G., Zizzo C., Dardis A., Bembi B., Cazzorla C., Rubert L., Zordan R. (2018). Newborn screening for lysosomal storage disorders by tandem mass spectrometry in North East Italy. J. Inherit. Metab. Dis..

[22] Burlina A.B., Polo G., Rubert L., Gueraldi D., Cazzorla C., Duro G., Salviati L., Burlina A.P. (2019). Implementation of Second-Tier Tests in Newborn Screening for Lysosomal Disorders in North Eastern Italy. Int. J. Neonatal Screen..

[23] Tanaka A., Sawada T., Suzuki K., Sakuraba H., Saito S., Sakabuchi T., Kitagawa T. Newborn screening of mucopolysaccharidosis I and II and characterization of pseudodeficiency alleles of iduronate 2-sulfatase gene found in the screening. Proceedings of the 12th International Symposium on MPS and Related Diseases.

[24] Ruijter G.J.G., Goudriaan D.A., Boer A.M., Van den Bosch J., Van der Ploeg A.T., Elvers L.H., Weinreich S.S., Reuser A.J. (2014). Newborn screening for hunter disease: A small-scale feasibility study. JIMD Rep..

[25] The UK NSC Recommendation on Mucopolysaccharidosis Type I. https://legacyscreening.phe.org.uk/mps1.

[26] Therrell B.L., Padilla C.D. (2018). Newborn screening in the developing countries. Curr. Opin. Pediatr..

[27] Borrajo G.J.C. (2007). Newborn screening in Latin America at the beginning of the 21st century. J. Inherit. Metab. Dis..

[28] Therrell B.L., Padilla C.D., Loeber J.G., Kneisser I., Saadallah A., Borrajo G.J.C., Adams J. (2015). Current status of newborn screening worldwide: 2015. Semin. Perinatol..

[29] Navarrete-Martínez J.I., Limón-Rojas A.E., Gaytán-García M.D.J., Reyna-Figueroa J., Wakida-Kusunoki G., Delgado-Calvillo M.D.R., Cantú-Reyna C., Cruz-Camino H., Cervantes-Barragán D.E. (2017). Newborn screening for six lysosomal storage disorders in a cohort of Mexican patients: Three-year findings from a screening program in a closed Mexican health system. Mol. Genet. Metab..

[30] Costa-Motta F.M., Acosta A.X., Abé-Sandes K., Bender F., Schwartz I.V.D., Giugliani R., Leistner-Segal S. (2011). Genetic studies in a cluster of mucopolysaccharidosis type VI patients in Northeast Brazil. Mol. Genet. Metab..

[31] Civallero G., Michelin K., de Mari J., Viapiana M., Burin M., Coelho J.C., Giugliani R. (2006). Twelve different enzyme assays on dried-blood filter paper samples for detection of patients with selected inherited lysosomal storage diseases. Clin. Chim. Acta.

[32] Giugliani R., Bender F., Couto R., Bochernitsan A., Brusius-Facchin A.C., Burin M., Amorim T., Acosta A.X., Purificação A., Leistner-Segal S. (2019). Population medical genetics: Translating science to the community. Genet. Mol. Biol..

[33] Camargo Neto E., Schulte J., Pereira J., Bravo H., Sampaio-Filho C., Giugliani R. (2018). Neonatal screening for four lysosomal storage diseases with a digital microfluidics platform: Initial results in Brazil. Genet. Mol. Biol..

[34] Bravo H., Neto E.C., Schulte J., Pereira J., Filho C.S., Bittencourt F., Sebastião F., Bender F., de Magalhães A.P.S., Guidobono R. (2017). Investigation of newborns with abnormal results in a newborn screening program for four lysosomal storage diseases in Brazil. Mol. Genet. Metab. Rep..

[35] Bender F., Burin M.G., Tirelli K.M., Medeiros F., Bitencourt F.H., Civallero G., Kubaski F., Bravo H., Daher A., Carnier V. (2020). Newborn screening for lysosomal disorders in Brazil: A pilot study using customized fluorimetric assays. Genet. Mol. Biol..

[36] Brusius-Facchin A.C., Siebert M., Leão D., Malaga D.R., Pasqualim G., Trapp F., Matte U., Giugliani R., Leistner-Segal S. (2019). Phenotype-oriented NGS panels for mucopolysaccharidoses: Validation and potential use in the diagnostic flowchart. Genet. Mol. Biol..

[37] Schwarz J.M., Cooper D.N., Schuelke M., Seelow D. (2014). MutationTaster2: Mutation prediction for the deep-sequencing age. Nat. Methods.

[38] Desmet F.-O., Hamroun D., Lalande M., Collod-Béroud G., Claustres M., Béroud C. (2009). Human Splicing Finder: An online bioinformatics tool to predict splicing signals. Nucleic Acids Res..

[39] Richards S., Aziz N., Bale S., Bick D., Das S., Gastier-Foster J., Grody W.W., Hegde M., Lyon E., Spector E. (2015). Standards and guidelines for the interpretation of sequence variants: A joint consensus recommendation of the American College of Medical Genetics and Genomics and the Association for Molecular Pathology. Genet. Med..

[40] Adzhubei I.A., Schmidt S., Peshkin L., Ramensky V.E., Gerasimova A., Bork P., Kondrashov A.S., Sunyaev S.R. (2010). A method and server for predicting damaging missense mutations. Nat. Methods.

[41] Venturi N., Rovelli A., Parini R., Menni F., Brambillasca F., Bertagnolio F., Uziel G., Gatti R., Filocamo M., Donati M.A. (2002). Molecular analysis of 30 mucopolysaccharidosis type I patients: Evaluation of the mutational spectrum in Italian population and identification of 13 novel mutations. Hum. Mutat..

[42] Polo G., Gueraldi D., Giuliani A., Rubert L., Cazzorla C., Salviati L., Marzollo A., Biffi A., Burlina A.P., Burlina A.B. (2020). The combined use of enzyme activity and metabolite assays as a strategy for newborn screening of mucopolysaccharidosis type I. Clin. Chem. Lab. Med..

[43] Tarini B.A., Goldenberg A.J. (2012). Ethical issues with newborn screening in the genomics era. Annu. Rev. Genomics Hum. Genet..

[44] Gelb M.H., Lukacs Z., Ranieri E., Schielen P.C. (2018). Newborn Screening for Lysosomal Storage Disorders: Methodologies for Measurement of Enzymatic Activities in Dried Blood Spots. Int. J. Neonatal Screen..

[45] Kubaski F., Mason R.W., Nakatomi A., Shintaku H., Xie L., van Vlies N.N., Church H., Giugliani R., Kobayashi H., Yamaguchi S. (2017). Newborn screening for mucopolysaccharidoses: A pilot study of measurement of glycosaminoglycans by tandem mass spectrometry. J. Inherit. Metab. Dis..

[46] Herbst Z.M., Urdaneta L., Klein T., Fuller M., Gelb M.H. (2020). Evaluation of Multiple Methods for Quantification of Glycosaminoglycan Biomarkers in Newborn Dried Blood Spots from Patients with Severe and Attenuated Mucopolysaccharidosis-I. Int. J. Neonatal Screen..

[47] Peck D.S., Lacey J.M., White A.L., Pino G., Studinski A.L., Fisher R., Ahmad A., Spencer L., Viall S., Shallow N. (2020). Incorporation of Second-Tier Biomarker Testing Improves the Specificity of Newborn Screening for Mucopolysaccharidosis Type I. Int. J. Neonatal Screen..

[48] Lajic S., Karlsson L., Zetterström R.H., Falhammar H., Nordenström A. (2020). The Success of a Screening Program Is Largely Dependent on Close Collaboration between the Laboratory and the Clinical Follow-Up of the Patients. Int. J. Neonatal Screen..

[49] Hall P.L., Sanchez R., Hagar A.F., Jerris S.C., Wittenauer A., Wilcox W.R. (2020). Two-Tiered Newborn Screening with Post-Analytical Tools for Pompe Disease and Mucopolysaccharidosis Type I Results in Performance Improvement and Future Direction. Int. J. Neonatal Screen..

